# Serial Cell-Culture Passage of Severe Fever with Thrombocytopenia Syndrome Virus Attenuates Virulence and Confers Protective Immunity in Mice

**DOI:** 10.3390/v18030333

**Published:** 2026-03-08

**Authors:** Jihee Kim, Young-Eui Kim, Hae Ji Kang, Jungsang Ryou, Hyuk Chu, Seok-Min Yun

**Affiliations:** Division of Acute Viral Diseases, Center for Emerging Virus Research, National Institute of Infectious Diseases, National Institute of Health, Korea Disease Control and Prevention Agency, Cheongju-si 28159, Republic of Korea; wlgml0721@korea.kr (J.K.); wlsdml7@korea.kr (Y.-E.K.); haeji.kang@korea.kr (H.J.K.); zenith87@korea.kr (J.R.); chuhyuk@korea.kr (H.C.)

**Keywords:** severe fever with thrombocytopenia syndrome virus, attenuation, serial passage, live-attenuated vaccine, immunogenicity, protective efficacy

## Abstract

Severe fever with thrombocytopenia syndrome (SFTS) is an emerging tick-borne viral disease with high case–fatality rates in East Asia, yet no licensed vaccines are currently available. Here, we generated attenuated severe fever with thrombocytopenia syndrome virus (SFTSV) strains through serial passage in Huh-7 cells and evaluated their pathogenicity, immunogenicity, and protective efficacy. Attenuation candidates were selected based on reduced pathogenicity, estimated based on the median lethal dose (LD_50_), and genetic sequencing was performed to identify mutations associated with attenuation. In C57BL/6 IFNAR^−^/^−^ mice, the attenuated strain exhibited markedly reduced virulence and viral loads while inducing robust virus-specific IgG, neutralizing antibody, and cellular immune responses. Notably, immunization with the attenuated strain conferred complete protection against lethal challenge with heterologous SFTSV genotypes. Genomic analysis revealed nonsynonymous mutations in the RNA-dependent RNA polymerase (RdRp), glycoprotein, and NSs genes, implicating alterations in viral replication, entry, and immune evasion. Collectively, these findings demonstrate that serial cell-culture passage can generate attenuated SFTSV strains that retain strong immunogenicity and cross-protective efficacy, supporting their potential as live-attenuated vaccine candidates for SFTS.

## 1. Introduction

Severe fever with thrombocytopenia syndrome (SFTS) is an emerging tick-borne infectious disease caused by the SFTS virus (SFTSV; recently renamed *Bandavirus dabieense*), a member of the genus *Bandavirus* in the family *Phenuiviridae* [1]. Since its first identification in China in 2009 [2], SFTS has been reported across multiple Asian regions, including South Korea, Japan, Vietnam, Taiwan, Pakistan, Myanmar, and Thailand, with case–fatality rates ranging from 10% to 30% [3,4,5,6,7,8,9,10,11,12,13]. Clinically, SFTS is characterized by high fever, gastrointestinal symptoms, leukopenia, thrombocytopenia, and multi-organ dysfunction, frequently leading to severe outcomes [2]. Despite its increasing public health significance, no licensed vaccines or specific antiviral therapies are currently available, highlighting the urgent need for effective countermeasures.

Similar to other viruses belonging to the family *Phenuiviridae*, SFTSV is a tri-segmented, single-stranded RNA virus with a negative and ambisense genome. A tripartite RNA genome comprises three segments of different sizes: large (L), medium (M), and small (S) segments; these three segments encode the RNA-dependent RNA polymerase (RdRp); the viral envelope glycoprotein (Gn and Gc); and a nucleocapsid protein (N) and a nonstructural protein (NSs), respectively [2,14].

Attenuation of pathogenic viruses through serial passage in cell culture has historically played a pivotal role in the development of live-attenuated vaccines, where cell culture adaptation reduced viral virulence while maintaining immunogenicity; classic examples include yellow fever, polio, and Japanese encephalitis vaccines [15,16,17]. This strategy relies on the accumulation of adaptive mutations generated during repeated replication in non-native host environments, which can alter viral tropism, replication efficiency, and immune recognition. Owing to the genetic and phenotypic plasticity of bunyaviruses, serial passage in cell lines may enable effective attenuation of SFTSV for vaccine development.

Previous studies have identified several viral determinants associated with SFTSV pathogenicity, including specific amino acid residues within the glycoproteins Gn/Gc and the nonstructural protein NSs, which modulate viral entry, replication, and antagonism of host innate immunity [18,19,20,21,22,23,24,25]. However, direct application of these molecular insights to vaccine design remains limited. In this context, systematic exploration of cell-culture-adapted attenuated strains can provide an empirical and translational pathway toward vaccine design. Moreover, the safety and efficacy of vaccine candidates can be confirmed through a comprehensive evaluation of replication kinetics, genomic changes, and immunogenicity of attenuated strains in vitro and in vivo.

In the present study, we aimed to establish an attenuated SFTSV strain through serial cell-culture passage and biologically characterize it. We investigated the replication capacity, genomic mutations, and immunogenicity of attenuated strains, as well as their protective efficacy in animal models. This study can potentially provide important insights into the effectiveness of cell-culture-mediated attenuation as a strategy for developing SFTSV vaccine candidates.

## 2. Materials and Methods

### 2.1. Ethics Statement

All animal experiments were conducted in an enhanced biosafety level 3 (BSL-3) laboratory following the guidelines of the Institutional Animal Care and Use Committee (IACUC) of the Korea Disease Control and Prevention Agency (approval number, KDCA-IACUC-24-006).

### 2.2. Cells and Virus

Vero E6 (African green monkey kidney epithelial cells; ATCC CRL-1586) and Huh-7 cells (human hepatocellular carcinoma cells; ATCC CRL-10741) were maintained in Dulbecco’s modified Eagle medium (DMEM, Gibco, Thermo Fisher Scientific, Waltham, MA, USA) and Rosewell Park Memorial Institute 1640 (RPMI 1640, Gibco, Thermo Fisher Scientific, Waltham, MA, USA) supplemented with 10% heat-inactivated fetal bovine serum (FBS, Gibco, Thermo Fisher Scientific, Waltham, MA, USA), penicillin-streptomycin (10,000 U/mL) (Gibco, Thermo Fisher Scientific, Waltham, MA, USA) at 37 °C with 5% CO_2_. In 2014, the wild-type SFTSV strain (KASJH, genotype D) was isolated from the serum of a Korean patient with SFTS [26] and propagated in Vero E6 cells. Viral titers were determined via focus-forming assay (FFA) using anti-SFTSV nucleoprotein (NP) antibody as previously reported [27].

### 2.3. Attenuation via Serial Passage

To induce attenuation, the parental SFTSV strain was serially passaged through Huh-7 cells. For each passage, cells were infected at a multiplicity of infection (MOI) of 0.1 and incubated at 37 °C for 5 days. Supernatants were harvested, clarified through centrifugation (3000 rpm for 5 min), and stored at −70 °C for subsequent passages. The attenuation was assessed by comparing in vitro and in vivo characteristics of viruses at the 10th, 20th, 30th, and 40th passages after 40 serial passages.

### 2.4. Viral Growth Kinetics and Protein Synthesis in Cell Culture

Subconfluent Huh-7 cell monolayers grown in 24-well plates were infected with wild-type (WT) and passaged viruses (MOI = 0.01), followed by incubation at 37 °C with 5% CO_2_. Supernatants were harvested daily for 7 days and stored in aliquots at −70 °C for further titration. The titers of SFTSV in daily samples were determined by FFA on Vero E6 cells. Moreover, cells harvested from the above mentioned daily samples were used to assess growth kinetics. Cell lysates were prepared and subjected to Western blotting at the same time points to quantify the expression of viral N and NSs proteins as described previously [27].

### 2.5. Genomic Sequencing

Viral RNA was extracted from cull culture supernatants of the WT, passage-10 (p10), passage-20 (p20), passage-30 (p30), and passage-40 (p40) SFTSV strains using the QIAamp Viral RNA Mini Kit (Qiagen, Hilden, Germany). To identify sequence differences between WT and passaged viruses, we sequenced the open reading frames (ORFs) of these viruses using the Sanger method following previously described methods [28].

### 2.6. Determination of LD_50_ of Serially Passaged Viruses

To assess the virulence of cell culture-adapted SFTSV strains, we determined the median lethal dose (LD_50_) for mice lacking the type 1 interferon receptor (Seven-to-Ten-week-old male C57BL/6 IFNAR^−^/^−^). Viral stocks were prepared from the 20th, 30th, and 40th passages in Huh-7 cells. A group of four mice was intramuscularly inoculated with 100 μL of 10-fold serial dilutions of each virus (10^−2^–10 FFU), with dilutions prepared in DMEM. The changes in body weight and survival of mice were monitored daily for 14 days. LD_50_ values were calculated via the Reed-Muench method [29] and compared among different passage-derived viruses.

### 2.7. Immunization and Sample Collection

To evaluate the immunogenicity of the attenuated strain, groups of C57BL/6 IFNAR^−^/^−^ mice (10–11-week-old male, *n* = 3 per group) were intramuscularly immunized with 10 FFU in 100 μL of either the attenuated p40 strain identified in this study, or a recombinant virus generated using reverse genetics (RG) based on the Korean isolate [27], in which the NSs gene was deleted, as previously reported [23,30]. A mock group (DMEM) served as a negative control. Blood samples were collected at week 3, and splenocytes were harvested for cellular assays.

### 2.8. Immunogenicity Assay

Sera from infected animals were analyzed for SFTSV-specific IgG antibodies through an in-house enzyme-linked immunosorbent assay (ELISA) using recombinant Gc antigen (r-Gc, SNB, Gwangmyeong, Gyeonggi, Korea). For this assay, Polysorp ELISA plates (Thermo Fisher Scientific, Waltham, MA, USA) were coated overnight with 0.1 ug/well of r-Gc at 4 °C. After incubation, the plates were blocked with 1% bovine serum albumin (BSA, Sigma-Aldrich, St. Louis, MO, USA) in phosphate-buffered saline (PBS, Gibco, Thermo Fisher Scientific, Waltham, MA, USA) and incubated for 1 h at room temperature (RT). After washing, the plates were incubated with 100 µL of four-fold diluted heat-inactivated serum with 1% BSA in PBS for 1 h at RT. The plates were washed three times with PBS containing 0.05% Tween-20 (PBST), incubated with horse radish peroxidase (HRP)- conjugated anti-mouse IgG antibody (Ab Frontier, Baileys Harbor, WI, USA) diluted with 1% BSA in PBS for 90 min at RT, and washed seven times. To detect antibodies, the plates were overlaid with O-phenylenediamine dihydrochloride (Sigma-Aldrich, St. Louis, MO, USA) substrate, and 0.5 M sulfuric acid (GenDEPOT, Baker, TX, USA) was added to block the reaction. Optical density (OD) at 490 nm was measured using a Multiskan SkyHigh microplate spectrophotometer (Thermo Fisher Scientific, Waltham, MA, USA).

Furthermore, mouse sera were collected 3 weeks after immunization, and neutralizing antibody was measured based on the 50% focus reduction neutralization titer (FRNT_50_). The KASJH strain in Vero E6 cells was used as a positive control. Briefly, 80 FFU of the KASJH strain was mixed with serially diluted inactivated sera and incubated for 1 h at 37 °C; subsequently, it was further inoculated into confluent monolayers of Vero E6 cells for 1 h at 37 °C. The same experimental procedure was applied to SFTSV strains representing other genotypes. The inocula were removed, and the medium was replaced with DMEM containing 10% FBS and 0.5% methylcellulose (Sigma-Aldrich, St. Louis, MO, USA). Cells were incubated at 37 °C for 2 days and fixed using 10% formaldehyde in PBS. Fixed plates were treated with 0.2% Triton X-100 (Sigma-Aldrich, St. Louis, MO, USA). After washing with PBS, cells were incubated with mouse anti-SFTSV NP monoclonal antibody (6B3, in-house) and then HRP-conjugated anti-mouse IgG antibody. The foci of infected cells were visualized using KPL TrueBlue™ peroxidase substrate (SeraCare Life Science, Milford, MA, USA). The FRNT_50_ values were calculated as the reciprocal of the highest dilution at which the number of foci was <50% of the number achieved without serum.

An enzyme-linked immunospot assay (ELISpot) was performed to evaluate the antigen-specific cellular immune responses induced by immunization. For this assay, spleens of immunized mice were harvested and immediately placed in RPMI 1640 medium supplemented with penicillin–streptomycin (10,000 U/mL). Splenocytes were mechanically dissociated using a tissue dissociator and passed through a 40-µm cell strainer (Corning, Corning, NY, USA), followed by centrifugation. Next, red blood cells were lysed by incubating them with 5 mL of RBC lysis buffer (Sigma-Aldrich, St. Louis, MO, USA), with gentle inversion, for 10 min at 37 °C in a 5% CO_2_ incubator. Subsequently, cells were washed and resuspended in RPMI 1640 containing 10% heat-inactivated FBS and penicillin-streptomycin, followed by centrifugation at 1500 rpm for 10 min. Cell pellets were resuspended in complete culture medium, and viable cells were counted using 0.4% trypan blue staining. Mouse IFN-γ and IL-4 ELISPOT assays (R&D Systems, Minneapolis, MN, USA) were performed following the instructions provided by the manufacturer. Before cell seeding, ELISpot plates were equilibrated with 200 µL of culture medium per well for 20 min at RT. Splenocytes were plated at different densities depending on the stimulation conditions: For positive control stimulation, splenocytes were seeded at 1 × 10^6^ cells per well and stimulated with phorbol 12-myristate 13-acetate (PMA, 50 ng/mL) and calcium ionomycin (0.5 µg/mL) (Invitrogen; Thermo Fisher Scientific, Waltham, MA, USA). For antigen-specific stimulation, splenocytes were seeded at 1 × 10^5^ cells per well and stimulated by incubating them with Gn protein peptide pool (1 µg/mL each peptide) overnight at 37 °C in a 5% CO_2_. Unstimulated cells cultured in medium alone served as negative controls. Subsequently, incubated plates were washed three times, and the appropriate biotinylated detection antibody mixture was added to each well and incubated for 2 h at RT. After additional washing steps, streptavidin–alkaline phosphatase conjugate was added and incubated for 2 h at RT. The spot was developed by adding BCIP/NBT substrate solution to each well, followed by incubation for 1 h at RT in the dark. The reaction was terminated by thorough washing with distilled water, and plates were air-dried overnight. Cytokine-secreting cells were visualized and quantified using a CTL ImmunoSpot Analyzer (ImmunoSpot, Cleveland, OH, USA).

### 2.9. Protective Efficacy

Groups of C57BL/6 IFNAR^−^/^−^ mice (10–11-week-old male, n = 5 per group) were immunized intramuscularly with either the attenuated p40 strain or a RG-derived NSs deletion mutant (NSs△2-282) (10 FFU per 100 μL). The viral inocula were diluted using DMEM to the desired concentration. Control mock-infected mice were inoculated with DMEM via the same route. At week 3 post immunization, mice were challenged intramuscularly with 10 FFU of lethal WT SFTSV strains belonging to the three different genotypes [B (KADGH strain), D (KASJH strain), and F (15KS8 strain)]. Mice were monitored daily for 14 days, focusing on changes in their body weight and survival. To examine the viral loads in serum and tissues (spleen, liver, and kidney), samples were collected from euthanized mice in groups of three, 3, and 5 days post-challenge (dpc). For virus titration in sera and homogenized tissues, infectious viral titers and viral RNA copy number were determined via FFA on Vero E6 cells and quantitative real-time RT-PCR (qRT-PCR), respectively, following previously described methods [27]. For histopathological analysis, tissue samples were collected from mice at designated time points post-challenge and immediately fixed in 10% neutral-buffered formalin for at least 24 h. Fixed tissues were processed using standard histological procedures, embedded in paraffin, and 3-µm-thick sections were prepared and stained using hematoxylin and eosin (H&E).

### 2.10. Statistical Analyses

Data were statistically analyzed using GraphPad Prism (version 8.2.1, GraphPad Software, La Jolla, CA, USA). The in vitro growth kinetics, antibody responses, and viral load in serum and tissues were compared using unpaired Student’s test or two-way analysis of variance (ANOVA) with Tukey’s or Sidak’s multiple comparisons test, as appropriate. Survival curves were evaluated using the log-rank (Mantel–Cox) test. *p*-values < 0.05 were considered statistically significant.

## 3. Results

### 3.1. In Vitro Characterization

**Figure 1:**
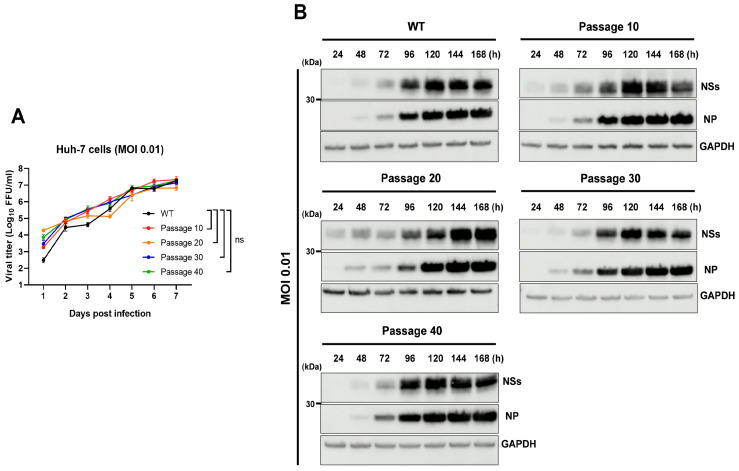
Comparison of in vitro growth kinetics and protein expression of the WT and serially passaged SFTSVs. (**A**) Growth kinetics of Huh-7 cells infected with parental, p10, p20, p30, and p40 viruses (MOI = 0.01). Culture supernatants were harvested from the infected cells at the indicated days after infection, and viral titers were determined by focus-forming assay using Vero E6 cells. Error bars indicate standard deviations (s.d.) of the mean. Statistical significance between the parental and passaged virus-infected groups was determined by an unpaired, two-tailed *t*-test; ns, not significant. (**B**) Viral protein expression in Huh-7 cell lysates was analyzed by immunoblotting using anti-SFTSV NP, anti-SFTSV NSs, and anti-GAPDH antibodies. GAPDH was used as the loading control.

To examine whether serial passage affected viral replication properties, we compared the in vitro characteristics of the wild-type (WT) and passaged viruses. Multi-step analysis of growth in Huh-7 cells revealed similar replication kinetics across all strains, with no significant differences in peak titers or replication rates. Likewise, viral protein expression patterns assessed by immunoblotting were comparable among the WT, passage-10 (p10), passage-20 (p20), passage-30 (p30), and passage-40 (p40) viruses. These results indicate that serial passage did not considerably alter the replication capacity or protein expression of the viruses in vitro (Figure 1).

### 3.2. Comparative LD_50_ of Serially Passaged Viruses

**Figure 2:**
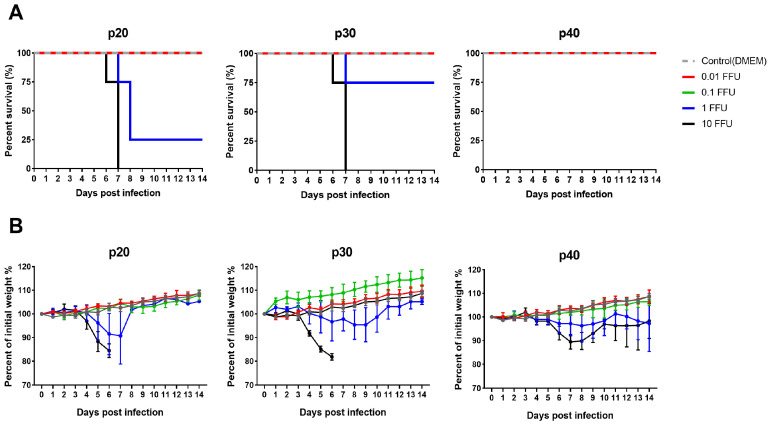
Determination of the median lethal dose (LD_50_) of serially passaged SFTSVs in C57BL/6 IFNAR^−^/^−^ mice. Groups of four mice were inoculated intramuscularly with 10-fold serial dilutions of each virus (10^−2^–10 FFU) and then monitored for survival rates (**A**) and body weight changes (**B**). Error bars indicate standard deviations of the mean.

Despite the similarity in in vitro profiles, serial passage in cell culture gradually attenuated SFTSV, as demonstrated through intramuscular inoculation of C57BL/6 IFNAR^−^/^−^ mice. The WT strain exhibited a low LD_50_, consistent with high virulence, while p20 and p30 viruses displayed moderately increased LD_50_ values. Notably, the highest dose (10 FFU) of p40 virus did not cause any mortality, which indicates a complete loss of lethality (Figure 2). These findings suggest that extended serial passage in Huh-7 cells induced a marked reduction in virulence, with p40 virus representing a highly attenuated phenotype.

### 3.3. Complete ORF Sequence Analysis of Serially Passaged Viruses

Comparative genomic analysis revealed the accumulation of nucleotide substitutions across L, M, and S segments with increasing passage number. As summarized in Table 1, only a limited number of synonymous mutations were observed in the p30 virus, while other viruses carried nonsynonymous substitutions, particularly within the RdRp, glycoprotein, and NSs genes. The emergence of amino acid changes in the p40 strain may be associated with the complete loss of lethality in vivo, highlighting potential molecular determinants of attenuation.

### 3.4. Humoral and Cellular Immune Responses in Mice

**Figure 3:**
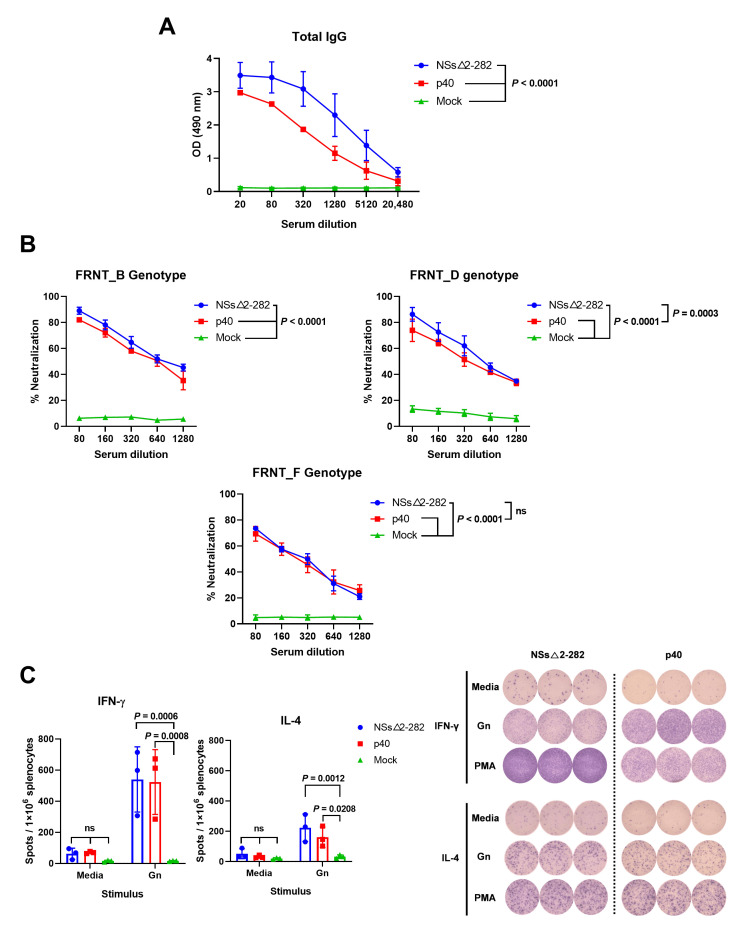
Humoral and cellular immune responses induced by p40 virus in C57BL/6 IFNAR^−^/^−^ mice. (**A**) Total anti-SFTSV IgG antibody titers measured by in-house ELISA using recombinant Gc antigen at 3 weeks post-immunization. (**B**) Neutralizing antibody titers against SFTSV strains representing three distinct genotypes (B, D and F) were determined by FRNT at 3 weeks post-immunization. Data represent mean ± s.d. (*n* = 3 per group). Statistical significance was determined using two-way ANOVA with Tukey’s multiple comparisons test. (**C**) The cellular immune response was detected via ELISpot. Representative images and spots of IFN-γ and IL-4 are presented. Statistical significance was determined by two-way ANOVA with Sidak’s multiple comparisons test. ns, not significant.

We compared the immunogenicity of the attenuated p40 strain and the NSs△2-282 mutant via immunization of C57BL/6 IFNAR^−^/^−^ mice and evaluation of antigen-specific humoral and cellular immune responses. Total IgG antibody titers were measured by ELISA, neutralizing activity was assessed by FRNT, and T cell responses were analyzed by IFN-γ and IL-4 ELISpot assays (Figure 3). ELISA analysis using recombinant SFTSV Gc protein demonstrated that both the attenuated p40 strain and the NSs△2-282 mutant induced robust virus-specific total IgG responses following immunization (Figure 3A). Notably, mice immunized with NSs△2-282 mutant exhibited IgG titers comparable to or slightly higher than those achieved using the p40 virus, whereas mock-immunized controls showed no detectable SFTSV-specific antibodies. Consistently, FRNT analysis using SFTSVs of different genotypes revealed that sera acquired from NSs△2-282 immunized mice exhibited strong neutralizing activity against SFTSV (Figure 3B). In contrast, p40-immunized mice developed detectable neutralizing antibodies, but at relatively lower titers. No neutralizing activity was observed in sera collected from mock-immunized mice. Virus-specific T cell responses were evaluated by ELISpot following stimulation with SFTSV-derived peptides. IFN-γ ELISpot assays revealed that both strains induced significant antigen-specific IFN-γ-secreting T cell responses compared with mock controls (Figure 3C). Based on IL-4 ELISpot analysis, we detected IL-4-producing cells in both groups, indicating induction of antigen-specific Th2 responses. However, IL-4 responses were lower than IFN-γ responses, and the IFN-γ/IL-4 ratio suggested a Th1-biased immune profile in both groups. These results indicate that both p40 and NSs△2-282 mutant strains induced balanced humoral and cellular immune responses in C57BL/6 IFNAR^−^/^−^ mice.

### 3.5. Cross-Genotype Protective Efficacy Against Lethal SFTSV Challenge

**Figure 4:**
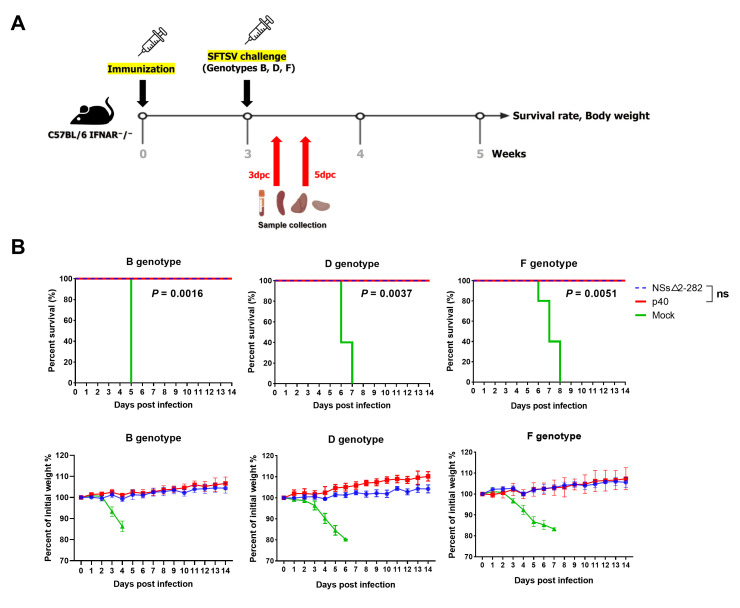
Cross-genotype protective efficacy against lethal challenge. (**A**) Schematic overview of the experimental design for protective efficacy analysis. (**B**) Survival rates and body weight changes following challenge with different SFTSV genotypes. Statistical differences in survival curves compared to mock-immunized control mice were calculated using a Log-rank (Mantel–Cox) test. ns, not significant.

All mock-immunized control mice succumbed to infection within 5–8 days after challenge with the three SFTSV genotypes. However, the attenuated p40 and the NSs△2-282 mutant immunized groups exhibited 100% survival, indicating complete protection against genotypes B, D and F challenge (Figure 4B).

**Figure 5:**
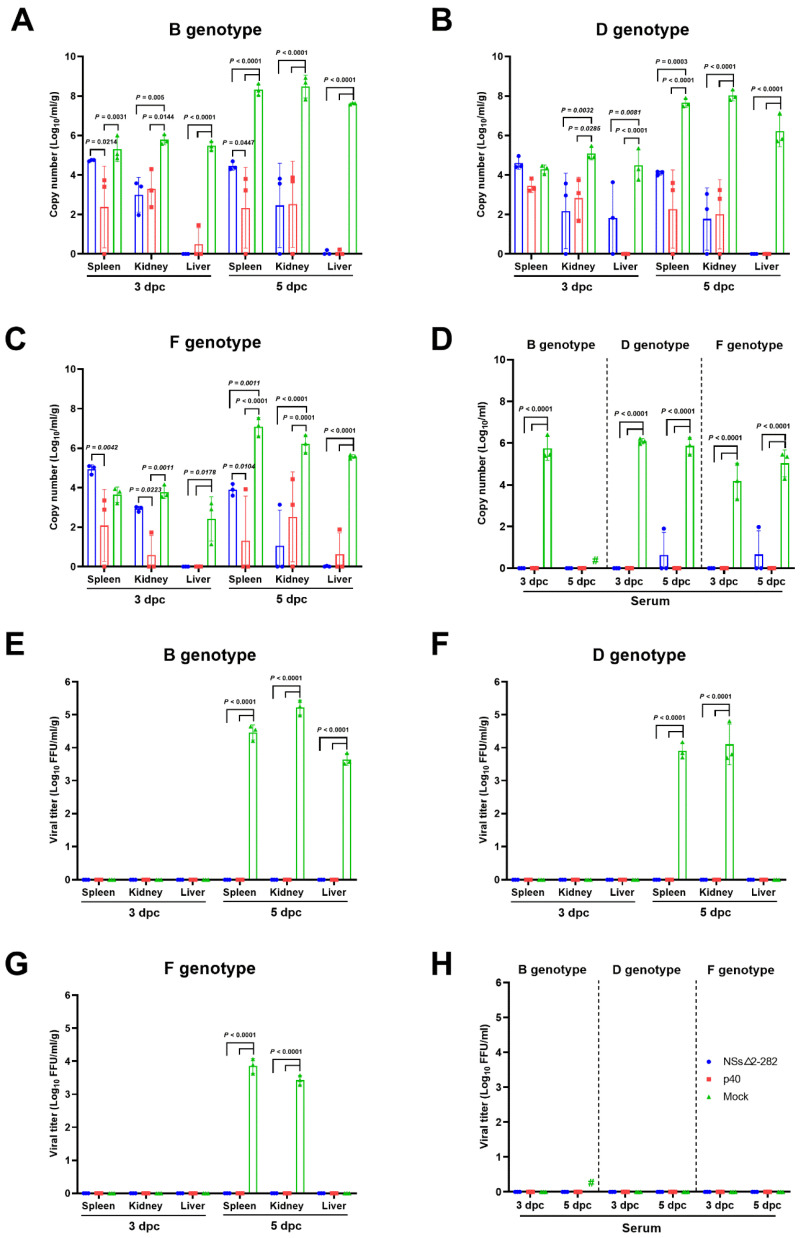
Viral load in tissues and serum of C57BL/6 IFNAR^−^/^−^ mice after heterologous SFTSV challenge. Viral RNA level and titers in various tissues (spleen, kidney, and liver) (**A**–**C** and **E**–**G**) and serum (**D**,**H**) at 3 and 5 dpc. Copy number and titers were determined through real-time qRT-PCR and focus-forming assay, respectively. Data are represented as the mean ± s.d. Statistical significance was determined using two-way ANOVA with Tukey’s multiple comparisons test. The sharp (#) indicates the unavailability of samples due to the death of all mice in this group.

To further analyze cross-genotype protective immunity induced by the attenuated p40 and the NSs△2-282 mutant strains, C57BL/6 IFNAR^−^/^−^ mice were challenged with lethal SFTSV genotypes B, D, and F, and viral loads in serum and tissues were quantified at 3 and 5 dpc. As shown in Figure 5, tissues and serum of mock-immunized mice exhibited a high level of viral replication across spleen, kidney, and liver, reaching approximately 10^2^–10^5^ copies/mL/g at 3 dpc and increasing to 10^6^–10^8^ copies/mL/g by 5 dpc. In contrast, other groups demonstrated profound suppression of viral loads. Mice p40 immunization reduced viral RNA levels in all tissues at 3 and 5 dpc. NSs△2-282 mutant-immunized mice also exhibited strong suppression of viral RNA; however, low-level residual RNA was detected more frequently than in the p40 group, particularly in the spleen (Figure 5A–C). A similar pattern was observed in serum viral RNA levels; mock-immunized mice exhibited high RNAemia (10^6^ copies/mL), whereas p40-immunized mice exhibited complete suppression across all viral genotypes and timepoints. The NSs△2-282 mutant induced near-complete suppression, with occasional minimal RNAemia in a small subset of genotypes D and F challenged mice (Figure 5D). Infectious viral titers in tissues measured by FFA confirmed these findings. High infectious titers (10^4^–10^5^ FFU/mL/g) were detected in tissues of mock-immunized mice at 5 dpc, with the highest loads in spleen and kidney. No infectious virus was detected in any tissues from other groups at 3 or 5 dpc (Figure 5E–G). In contrast to tissue infectivity, infectious viral titers in serum were not detected in any group, including mock, p40, or NSs△2-282 at 3 and 5 dpc (Figure 5H). Although mock-immunized mice exhibited high viral RNAemia, no infectious particles could be recovered from the serum.

**Figure 6:**
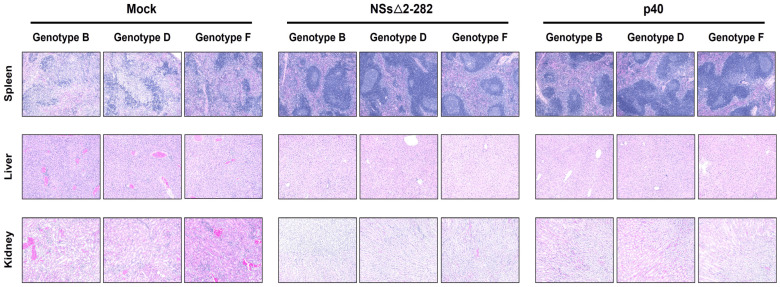
Histopathological analysis of tissues following heterologous SFTSV challenge. Hematoxylin and eosin (H&E)-stained sections of spleen, liver, and kidney tissues collected at 5 dpc from C57BL/6 IFNAR^−^/^−^ mice immunized with Mock, NSs△2-282, or p40 and subsequently challenged with SFTSV belonging to different genotypes (B, D and F). Magnification, 100×.

Next, histopathological analysis of spleen, liver, and kidney tissues, collected at 5 dpc, revealed the extent of tissue damage following heterologous SFTSV challenge. In mock-immunized mice challenged with SFTSV genotype B, D, and F, severe histopathological lesions were consistently identified across all examined organs (Figure 6). Pronounced disruption of splenic architecture, including depletion of white pulp and expansion of red pulp areas, indicated severe lymphoid damage in the spleen. The liver exhibited extensive hepatocellular degeneration and necrosis, accompanied by marked inflammatory cell infiltration and focal hemorrhage. In the kidney, tubular epithelial cell degeneration and interstitial inflammation were evident, reflecting systemic viral dissemination and organ injury. In contrast, mice immunized with the attenuated p40 and the NSs△2-282 mutant strains exhibited minimal to no histopathological abnormalities following challenge with any SFTSV genotype. These findings demonstrate that both vaccine candidates effectively prevented SFTSV-induced tissue pathology across multiple viral genotypes.

## 4. Discussion

The present study demonstrated that serial passage of SFTSV in Huh-7 cells progressively reduced viral pathogenicity in vivo, as reflected by increased LD_50_ values across successive passages. Importantly, the p40 virus did not cause mortality even at the highest inoculum tested, suggesting a fully attenuated phenotype induced by extended cell culture adaptation. These results are consistent with the classical examples of live-attenuated vaccines developed through serial passage, such as the yellow fever 17D and the oral poliovirus vaccines, in which adaptive mutations accumulated during prolonged passage reduced the virulence while preserving immunogenicity [15,16]. The complete loss of lethality associated with the p40 strain indicated its potential as a candidate for live-attenuated vaccine development against SFTSV. Interestingly, comparative in vitro analyses revealed no significant differences in viral growth kinetics or protein expression patterns among the parental and passaged viruses, indicating that the viral attenuation was not attributable to impaired replication capacity or defective viral protein synthesis in cell culture. Instead, our findings suggest that specific genetic changes acquired during prolonged passage have potentially altered viral determinants of virulence in vivo, while preserving fundamental replication processes in vitro. Previously, similar discrepancies between in vitro and in vivo phenotypes were reported in SFTSV [23,25,31,32]. Overall, the p40 strain represents a highly attenuated phenotype with preserved replication fitness, supporting its potential as a live-attenuated vaccine candidate.

Genomic sequencing of the attenuated strain revealed nonsynonymous mutations in the RdRp genes, the glycoprotein Gn, and the nonstructural protein NSs compared to the parental strain. Notably, despite comparable in vitro replication profiles, the p40 strain exhibited unique amino acid substitutions that coincided with a complete loss of lethality in vivo. Hence, the observed attenuation is likely attributable to specific genomic alterations affecting viral virulence determinants rather than general replication capacity. The integration of whole-genome sequence data with phenotypic characterization provides important insights into the molecular basis of attenuation. Further reverse genetic studies can validate the contribution of these mutations to the loss of virulence in the p40 strain, which may guide the development of live-attenuated vaccine candidates.

C57BL/6 IFNAR^−^/^−^ mice infected with the attenuated strain exhibited markedly improved survival and lower viral loads compared to those infected with the parental strain. Immunized mice developed strong SFTSV-specific immune responses, including virus-specific IgG antibodies, neutralizing activity, and antigen-specific T cell responses, and were fully protected against lethal challenge. Neutralization assays performed against heterologous SFTSV strains representing genotypes B and F revealed detectable neutralizing activity compared with the homologous genotype. These findings indicate that immunization with the attenuated p40 virus elicits antibodies with cross-neutralizing capacity, supporting the observed in vivo cross-protective efficacy. Notably, despite inducing lower neutralizing antibody titers than the NSs△2-282 mutant, immunization with the p40 virus resulted in complete protection against lethal challenge. This apparent dissociation between neutralizing antibody magnitude and protective efficacy suggests that additional immune mechanisms may contribute to vaccine-induced protection. One possibility is that limited yet detectable in vivo replication of the p40 virus enhances antigen presentation and promotes robust cellular immune responses, particularly Th1-biased T cell-mediated immunity. Th1-dominant responses, characterized by enhanced cellular immunity, are known to play a critical role in viral clearance and long-term protection, particularly in the context of genetically diverse SFTSV strains. Therefore, lower neutralizing antibody titers should not necessarily be interpreted as reduced protective potential. Rather, the p40 virus may induce a qualitatively broader and Th1-skewed immune response that compensates for quantitative differences in humoral immunity and contributes to effective protection. Comparably, attenuated strains of other bunyaviruses were reported to elicit durable immunity [33,34]. The ability of the attenuated strain to induce both protective humoral responses and cytokine production supports its potential as a live-attenuated vaccine candidate. To date, no licensed vaccines or antivirals are available for SFTS. Several approaches, including inactivated vaccines, subunit vaccines, and viral-vectored vaccines, are under development [35]; however, each has immunogenicity or safety-related limitations. Live-attenuated vaccines can mimic natural infection and generally elicit broader and longer-lasting immunity. Our findings suggest that cell-culture-mediated attenuation of SFTSV may present a practical path toward vaccine development. However, safety concerns, including the possibility of reversion to virulence, must be rigorously evaluated via preclinical and clinical studies.

The present study demonstrates that both the serially passaged attenuated p40 strain and the NSs△2-282 mutant confer potent cross-genotype protection against SFTSV infection in C57BL/6 IFNAR^−^/^−^ mice. The virological outcomes observed across serum and tissue compartments confirm that the induced immunity effectively prevents systemic viral dissemination following heterologous challenge. The absence of infectious virus in all immunized mice, as shown in Figure 5, highlights the robust antiviral protection mediated by both vaccine candidates. Despite high viral RNAemia in mock-immunized mice, infectious virus was not detectable in their serum, potentially due to rapid redistribution of infectious particles into tissues or early neutralization. Vaccinated animals showed complete suppression of infectious virus across all compartments. Consistently, the p40 virus induced stronger viral suppression compared with the NSs△2-282 mutant, which may be attributed to differences in attenuation mechanisms, antigen availability, or replication dynamics. Consistent with the virological findings, severe tissue damage was observed exclusively in mock-immunized mice, whereas vaccinated animals were largely protected from SFTSV-induced organ pathology. The extensive hepatic necrosis, splenic architectural collapse, and renal tubular injury in mock controls reflect hallmark pathological features of severe SFTSV infection correlated with high viral burdens and poor clinical outcomes. In contrast, the near-complete preservation of tissue integrity in p40 and NSs△2-282-immunized mice indicates effectively reduced viral replication and prevention of downstream immunopathology. This observation aligns closely with the undetectable infectious viral particles and considerable reduction in viral RNA loads in these animals. Hence, the histopathological data corroborate the survival, viral load, and immunogenicity results, demonstrating that vaccination with the p40 virus confers robust cross-genotype protection against SFTSV-induced tissue injury. These findings further support the potential of the p40 virus as a promising live-attenuated vaccine candidate, which can prevent viral replication and associated organ pathology.

The present study has limitations. First, attenuation was achieved in vitro, and limited in vivo models were used to confirm reduced pathogenicity. Further analyses involving additional animal models, such as ferrets or nonhuman primates, can validate the safety and immunogenicity of the vaccine candidate [23,36]. A key limitation of the present study is the exclusive use of C57BL/6 IFNAR^−^/^−^ mice for in vivo evaluation. While this model is widely used for SFTSV pathogenesis and vaccine studies because of its high susceptibility, the absence of type I interferon signaling represents a major deviation from immunocompetent hosts. Consequently, attenuation, safety, and immunogenicity profiles observed in this model may not fully reflect outcomes in hosts with intact innate immunity. Therefore, conclusions regarding vaccine safety and translational potential should be interpreted with caution. Further studies incorporating immunocompetent animal models are essential to further validate the attenuation phenotype and to better define the immune mechanisms underlying protection. Second, while mutations in RdRp, Gn, and NSs genes were identified, their functional roles in attenuation remain to be dissected using reverse genetics. Genetic analysis in the present study was based on Sanger sequencing of consensus ORF sequences to identify dominant mutations associated with attenuation. While this approach is suitable for detecting fixed, reproducible genetic changes, it does not capture low-frequency variants or intrapopulation diversity. Accordingly, future studies incorporating next-generation sequencing will be required to further assess genetic heterogeneity and long-term stability of the attenuated p40 strain. Third, despite the strong cross-genotype protective efficacy demonstrated in the present study, an important limitation is the unclear knowledge of long-term genetic stability and the potential for virulence reversion of the attenuated p40 strain. Although no pathogenicity was observed in the in vivo experiments presented in the present study, serially passaged live-attenuated viruses may theoretically accumulate compensatory mutations that could restore partial virulence under certain conditions. Therefore, comprehensive whole-genome sequencing during extended passaging, along with in vivo reversion assessment in multiple animal models, is required to confirm the long-term safety profile of the p40 strain as a vaccine candidate. Fourth, cellular immune responses in the present study were evaluated using a Gn-derived peptide pool, reflecting the established role of SFTSV glycoproteins as major targets of protective immunity. While this approach captures a relevant component of vaccine-induced T cell responses, we acknowledge that limiting stimulation to Gn peptides provides an incomplete assessment of the overall cellular immune repertoire. Other viral antigens, such as Gc, N, or NSs, may contribute additional and potentially broader T cell responses, including those relevant to cross-protection. Therefore, the observed Th1-biased response should be interpreted as indicative of glycoprotein-specific cellular immunity rather than a comprehensive measure of total cellular immune responses. Future studies incorporating multi-antigen peptide pools or whole-virus stimulation will be required to more fully define the breadth and correlates of cellular immunity elicited by the attenuated p40 virus. Finally, a large-scale evaluation of the protective efficacy and durability of immunity is crucial for further clinical translation.

## 5. Conclusions

In summary, this study establishes that serial cell-culture passage of SFTSV can attenuate the virulence of this virus while eliciting strong protective immunity. These results lay the foundation for the development of live-attenuated SFTSV vaccines and support the broader efforts to manage this emerging and highly fatal disease.

## Figures and Tables

**Table 1:** Variation in nucleotide and amino acid sequences between the WT and passaged SFTSVs.

Segment	Position *		Nucleotide Changes					Amino Acid Changes				
	NT	AA	WT	p10	p20	p30	p40	WT	p10	p20	p30	p40
L	4	2	A	G	G	G	G	N	D	D	D	D
	5463	1821	G	G	G	A	G	P	P	P	P	P
M (Gn)	98	33	A	A	A	A	C	N	N	N	N	T
S (NSs)	117	39	A	A	T	T	T	E	E	D	D	D

* Nucleotide and amino acid residue numbers are based on the complete ORF sequence of the KASJH strain, GenBank accession nos. KP663731–KP663733.

## Data Availability

The original contributions presented in this study are included in the article. Further inquiries can be directed to the corresponding author.
